# Impact of blood contamination on hydrophilic metabolomics in human meningioma tissue

**DOI:** 10.1007/s00216-026-06623-x

**Published:** 2026-07-01

**Authors:** Veronika Fitz, Simone Braeuer, Lisa Panzenboeck, Harald Schoeny, Daniela Loetsch, Nicole Bachhofner, Dorian Hirschmann, Gunda Koellensperger

**Affiliations:** 1 Faculty of Chemistry, Institute of Analytical Chemistry, University of Vienna, Vienna, Austria; 2 Vienna Doctoral School in Chemistry (DoSChem), University of Vienna, Vienna, Austria; 3 Institute of Analytical Chemistry, Department of Natural Sciences and Sustainable Resources, BOKU University, Vienna, Austria; 4 Department of Neurosurgery, Comprehensive Cancer Center, Medical University of Vienna, Vienna, Austria; 5 Vienna Metabolomics Center (VIME), University of Vienna, Vienna, Austria; 6 Chemistry Meets Biology, University of Vienna, Vienna, Austria

**Keywords:** Metabolomics, Elemental analysis, Blood contamination, Meningioma, Iron

## Abstract

Contamination of tissue samples with blood is hardly avoidable. The possibilities for replicate sampling and tissue washing are limited when working with resected specimens of human origin. However, its impact on metabolic profiling has rarely been addressed. In this study, we performed elemental analysis and metabolic profiling focusing on 108 hydrophilic target metabolites in 61 meningioma tissue samples obtained from a non-stratified cohort of 36 patients during neurosurgery. We used iron as an easily accessible proxy for blood. The samples were frequently and non-homogenously contaminated with blood, with iron concentrations ranging from 5 to > 160 µg/g fresh weight. Ten metabolites were significantly altered with their concentrations more than halved or more than doubled when comparing samples with low and high degrees of blood contamination. By contrast, blood contamination did not dominate the apparent metabolic heterogeneity between the samples during PCA analysis based on the metabolites targeted in this study and in the context of a heterogeneous patient cohort and non-standardized sampling. However, studies targeting the reported vulnerable metabolites should critically reflect blood contamination as a biasing factor. Moreover, we report estimated concentrations of 74 hydrophilic metabolites to give tissue-specific orientation for future quantification studies.

**Supplementary Information:**

The online version contains supplementary material available at 10.1007/s00216-026-06623-x.

## Introduction

Although it is widely recognized that metabolite leakage from red blood cells can distort the metabolic profiles of blood and cerebrospinal fluid samples [1, 2], the potential bias from blood contamination is seldom considered in tissue-based metabolomics [3, 4]. Tissue contamination with blood is difficult to avoid, and erythrocytes are likely to release intracellular metabolites during standard metabolite extraction procedures [5]. Washing excised tissue—a practice occasionally recommended to reduce blood contamination [6, 7]—removes only surface blood and is insufficient for highly vascularized specimens. Systematic strategies to quantify or correct for blood contamination are rarely implemented, raising the question of whether (and to which extent) blood presence confounds sample clustering and phenotype assignment.

Meningiomas are tumors arising from the middle layer of the meninges, the membranes surrounding the brain and spinal cord. With ~ 30% of the cases, they are the most common type of primary brain tumor [8]. Metabolic, cellular, and molecular heterogeneity within a single tumor is generally lower in meningioma than in highly malignant brain tumors such as glioblastoma. By contrast, different textures and resection behavior of meningiomas are observed during neurosurgery. Angiogenesis within and around meningiomas is common and depends on tumor grade and histological subtype [9], making uneven blood contamination in resected samples likely. In the context of metabolic profiling, heterogeneous degrees of blood contamination among the compared samples are particularly problematic for metabolites that are abundant in blood and at the same time biologically relevant to tumor metabolism—such as glucose, lactate, amino acids, or nucleotides. Here, contamination could obscure genuine tissue-specific metabolic signatures and spatial heterogeneity [4].

The middle layer of the meninges (the arachnoid mater), where meningiomas grow from, primarily consists of fibroblasts and connective tissue [10] and is not inherently rich in iron. At the same time, around 65% of the body’s total iron is contained in blood hemoglobin [11]. About 30% is found in specific organs like skeletal muscle, bone marrow, liver and macrophages of the reticuloendothelial system [12]. This specific distribution in the human body suggests iron as a surrogate marker for blood contamination in tissue.

Absolute quantification is increasingly recognized as essential for improving the utility, reproducibility, and interpretability of metabolomics data [13–15]. Absolute concentrations also support clinically relevant applications, where standardized reference ranges are required to validate biomarkers [16]. Achieving absolute quantification in MS-based metabolomics requires calibration using authentic standards at relevant concentration ranges [14]. Given that metabolic signatures differ between tissues—and between tumors and healthy tissue [17–19]—delineating the expectable range of concentrations in pilot data is critical for accurate tissue-specific calibration.

In this study, we address two underexplored challenges in tissue metabolomics—tumor heterogeneity and blood contamination—by analyzing human meningioma samples obtained during neurosurgery. We quantify the extent of blood contamination, characterize its variability both between patients and across samples from the same tumor, and evaluate its impact on metabolic profiles. We also provide estimated concentrations for a wide range of central carbon metabolites as a reference for meningioma tissue. To our knowledge, this is the first study to quantitatively examine blood contamination in human tissue metabolomics and assess its potential to bias profiling of the hydrophilic central carbon metabolome.

## Materials and methods

### Chemicals and standards

Acetonitrile, methanol, isopropanol, water, formic acid, and ammonium formate were of LC-MS grade and ordered at Fisher Scientific (Vienna, Austria) or Sigma Aldrich (Vienna, Austria). Nitric acid was obtained from Carl Roth (Rotipuran© Supra, 69%, Karlsruhe, Germany). Metabolite standards were purchased from Sigma Aldrich (Vienna, Austria) or Carbosynth (Berkshire, UK). All standards were weighed and dissolved in an appropriate solvent and volumetrically joined to obtain a mix of ~ 200 metabolites. The mix was aliquoted in HPLC vials, evaporated to dryness, and stored at −80 °C. One aliquot was reconstituted in 200 µL water, giving a multi-metabolite stock solution which was further diluted with isopropanol/water 7:3 (v/v) to obtain twelve calibrator concentrations for external calibration (0.005–25 µmol L^−1^). Element standards were purchased from Labkings B.V. (Hilversum, The Netherlands).

### Samples

Meningioma tissue samples of varying size were obtained from 36 patients after neurosurgery and stored at −80 °C. Most tumors were classified as WHO grade I, few as WHO grade II. Sixteen patients were diagnosed with peritumoral edema, 12 with varying forms of seizures, and 8 exhibited edema and seizures. Larger tumor samples were cut into adjacent pieces before homogenization: They are referred to as technical replicates from the same tumor or patient in the following.

#### Tissue homogenization

The samples were divided into three batches for homogenization. Large tumor samples were cut with a surgery knife on a glass slide on dry ice before being weighed: 13 of the surgically obtained 36 tumors were cut into two pieces, 4 were cut into four pieces. Each of these pieces was homogenized, extracted and analyzed separately in the following. They are referred to as technical replicates from the same tumor or patient. Each tissue sample (50–120 mg fresh weight) was transferred to a homogenization tube pre-filled with 200 µL ice-cold methanol and quickly weighed. The tube was filled up with methanol to obtain a tissue concentration of 1 mg/8 µL and put back on dry ice. The homogenization tubes were transferred to a Precellys Evolution Touch homogenizer pre-cooled with liquid nitrogen (air temperature approx. −5 °C). The homogenization cycle comprised two 20 s bursts at 10,000 rpm with a 30 s pause in between. The samples were homogenized with a total of 3 cycles and put back on dry ice. For each sample, an 80 µL aliquot corresponding to ~ 10 mg fresh weight was placed in an Eppendorf tube on dry ice (using a 0.1–1 mL-pipette to avoid clogging of the tip) and stored at −80 °C until extraction.

#### Metabolite extraction

A total of 61 homogenates were analyzed. 120 µL acetonitrile/water 2:1 (v/v) was added to reach a solvent composition of acetonitrile/methanol/water 2:2:1 (v/v/v). The samples were vortexed for 10 s and put on wet ice for 15 min. Thereafter, the samples were spun at 20,000 rcf and 4 °C for 10 min to pellet the precipitated protein. The supernatants were transferred to new Eppendorf tubes and evaporated to dryness in a vacuum centrifuge for ~ 4 h at ~ 30 °C. The dried extracts were stored at −80 °C until further analysis. The wet pellets were stored at −20 °C until further analysis.

#### Preparation of LC-MS samples

The samples were reconstituted in 300 µL isopropanol/water 7:3 (v/v) and transferred to brown-glass HPLC vials with 200 µL inserts for LC-MS analysis. A pooled QC sample was prepared for each homogenization batch by mixing 10 µL of each reconstituted sample. A global pooled QC sample was prepared by mixing the batch pools at equal amounts.

#### Blank

A procedural blank consisting of 100 µL water instead of tissue was prepared for each homogenization batch and taken through homogenization, aliquoting, extraction and measurement alongside the samples.

#### Digestion for elemental analysis

The protein pellets were dried in an incubator at 80 °C for 90 min. An empty Eppendorf tube served as digestion blank. 300 µL nitric acid and 400 µL purified water (resistivity at least 18 MΩ cm) were added to each sample, followed by shaking in an orbital shaker at 90 °C for 90 min. The samples were quantitatively transferred to Falcon tubes and filled up to 3 mL with water. The samples were stored at room temperature until elemental analysis. The standard reference material SRM 1577c (Bovine Liver, NIST, N = 1, trueness = 98%) was prepared and analyzed along with the samples.

### Multielement analysis

Iron concentrations were analyzed with inductively coupled plasma mass spectrometry (ICP-MS, 8800 ICP-QQQ, Agilent Technologies) on m/z 56 in helium collision gas mode. Quantification was achieved via external calibration with a Fe standard solution. For quality assurance, Ge was added online as internal standard to account for matrix effects and possible changes in instrument sensitivity.

### Metabolomics

#### Liquid chromatography

The metabolite extracts were analyzed using parallel HILIC (hydrophilic interaction liquid chromatography) and RP (reversed phase) separation. The same extract was injected and eluted from the HILIC column, followed by injection and elution from the RP column while the HILIC effluent was directed to waste using a switching valve during re-equilibration. HILIC separation was performed with an Acquity UPLC BEH Amide (2.1 × 150 mm, 1.7 μm, Waters) equipped with an Acquity UPLC BEH Amide VanGuard precolumn (5 × 2.1 mm, 1.7 μm, Waters) using water with 10 mM ammonium formate and 0.125% formic acid as eluent A and acetonitrile/water 95:5 (v/v) with 10 mM ammonium formate and 0.125% formic acid as eluent B. The injection volume was set to 5 μL and the flow rate to 400 μL/min for separation and re-equilibration. The following gradient was applied: 0–7.7 min linear decrease from 100 to 70% B and 7.7–10.25 min linear decrease to 30% B, 10.25–12.75 min increase to 100% B, 12.75–14 min flush at 100% B. The flow rate was reduced to 50 μL/min during RP separation (13–28 min) to save on eluents. RP separation was performed with an Acquity UPLC HSS T3 (2.1 mm × 150 mm, 1.8 μm, Waters) equipped with an Acquity UPLC HSS T3 VanGuard Pre-column (2.1 mm × 5 mm, 1.8 μm, Waters) using water with 0.1% formic acid as eluent A and 95% MeOH mixed with 5% eluent A as eluent B. The injection volume was set to 5 μL and the flow rate to 250 μL/min for separation and re-equilibration. The following gradient was applied: 13–24 min linear increase from 0 to 100% B, 24–28 min hold at 100% B, 28 min switch to 0% B, followed by 5 min re-equilibration to starting conditions. The flow rate was reduced to 50 μL/min during HILIC separation to save on eluents. The column compartment temperature was 45 °C. Samples and standards were kept in the autosampler at 4 °C. The injection needle was washed for 5 s with a mixture of acetonitrile, methanol, and water 1:1:1 (v/v/v) between the injections.

#### ESI-Orbitrap-HRMS

High-resolution mass spectrometry was performed on an Orbitrap IQ-X Tribrid mass spectrometer (Thermo Scientific) equipped with a heated electrospray ion source (H-ESI). The H-ESI source parameters were the following: collision gas pressure 1 mTorr, spray voltage 3.1 kV in both positive and negative mode, sheath gas 50 A.U., auxiliary gas 20 A.U., sweep gas 1 A.U., ion transfer tube temperature 300 °C, vaporizer temperature 350 °C, small molecule application mode, default charge state 1. Full mass scan data were acquired in profile mode in a scan range of 60–900 m/z. Positive and negative mode switching was applied with a resolution of 60,000 at m/z 200. The AGC target was set to 400,000 (100%), the RF lens to 30% and the maximum injection time was 118 ms (auto mode).

#### Data processing

The raw data were centroided and converted to mzML format with msConvert GUI (version 3.0.19014-f9d5b8a3b) [20]. Extracted ion chromatograms of ~ 150 target metabolites were then generated in Skyline [21] with a mass extraction window of 5 ppm. The evaluation focused on [M+H]^+^ and [M-H]^−^ adducts of the monoisotopic masses. The molecules, including isomers, were identified by retention time comparison with authentic standards. A.csv file stating area values, raw intensities, and mass error was further processed with R [22]. Molecules that were detected in at least 80% of the samples were considered for further analysis. The intensity values fell below the limit of detection in some samples. These missing values were replaced by haft of the lowest positively measured intensity for that metabolite to provide a complete set of values for unsupervised clustering. These metabolites were excluded from quantification in the respective samples. Despite careful normalization of the sample amounts based on fresh weight before extraction, we experienced different summed amounts of target metabolites, expressed as the total ion current of the targeted and positively identified metabolites per sample, mTIC [23]. The individual metabolite intensities were hence subjected to mTIC normalization [24]. Metabolites with missing intensities in any of the samples (two metabolites in positive mode and two in negative mode in the present data) were omitted from mTIC calculation.

#### Unsupervised clustering

Principal component analysis was performed on the mTIC-normalized data using the *prcomp()* function from the *stats* package (version 4.1.3) in R [22] after variable stability (vast) scaling [25]. Plots were generated using different target lists: first, a broad target list of 108 detected metabolites, including amino acids, nucleic acids and their derivatives, sugars and sugar phosphates, etc. (Table S1), was included to check the general clustering of the samples. As erythrocyte-derived metabolites have a strong influence on the metabolic profile of full blood due to their abundance and high metabolic activity [26], the clustering analysis was repeated with pathway-specific subsets of metabolites covering pentose phosphate pathway, anaerobic glycolysis, purine and nucleoside salvage pathway, and redox metabolism, which dominate red blood cell metabolism (Table S2) [27].

#### Quantification

The mTIC-normalized data were used for semi-quantification. The relationship of concentration and signal intensity was evaluated for > 100 molecules detected in at least 80% of the samples using a multi-component mix of external authentic standards at 14 concentrations spanning 0.001 to 25 µmol/L in neat solvent. Isomers lacking chromatographic baseline separation were summarized. The lower limit of quantification (LLOQ) was defined as 3× the signal intensity of the lowest calibrator in the linear range. Molecules with linear behavior over at least 3 calibrators that were detected ≥ LLOQ in at least 10 samples were considered for quantification. Depending on the median metabolite concentration over all samples, the best-matching calibrator concentration was used for one-point calibration. Molecules < LLOQ were omitted from quantification while molecules exceeding the linear range (> estimated upper limit of quantification, ULOQ) were quantified and commented in the report.

## Results

This study evaluates the impact of blood contamination on metabolomics biomarker discovery from clinical tissue samples. It is based on meningioma samples from 36 patients (one tumor per patient) obtained during neurosurgery. Including technical replicates, a total of 61 homogenates were analyzed.

### Blood contamination

During gross visual examination, the tumor homogenates appeared in color shades between off-white and dark red. To demonstrate the problem, photographs of three homogenates obtained from different patients are depicted in Figure S1A. The suspected differences in blood contamination were underlined by elemental analysis of the protein pellet via ICP-MS after metabolite extraction and acidic digestion: The iron concentrations of the samples ranged from 5 to more than 150 µg/g fresh weight (Table S3, Figure S1B). By contrast, iron concentrations were relatively stable within technical replicates of the same tumor with a maximum observed variation of 36% RSD (N = 4). Although iron concentration is a continuous variable, we empirically found that the samples formed clusters at iron concentrations below 25 µg/g and above 50 µg/g (Figure S1B), which we hence refer to as low or base iron concentration and high iron concentration, respectively.

### Differential metabolites

**Fig. 1:**
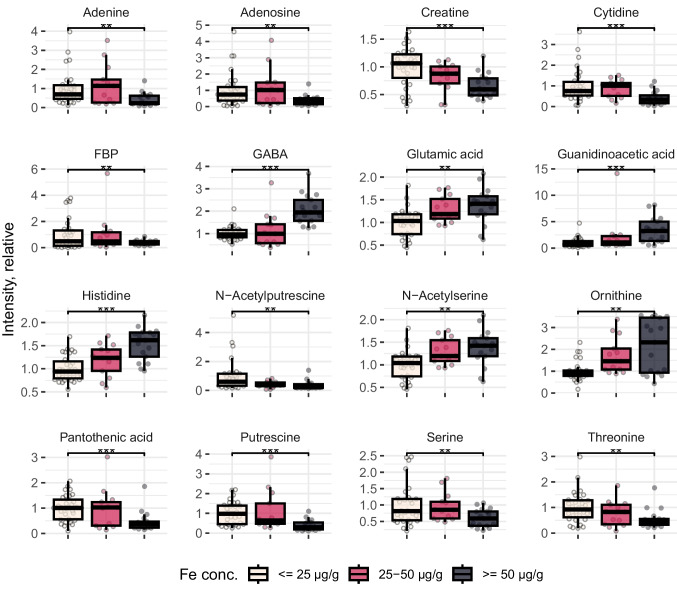
Significantly altered metabolites comparing low- and high-blood contamination samples. The y-axis shows intensities normalized to the mean intensity of the sample group with the lowest iron concentration. The scattered dots represent individual samples. The adjusted *p*-value is indicated with asterisks: *p ≤ 0.05, **p ≤ 0.01, ***p ≤ 0.001. FBP: Fructose-1,6-bisphosphate, GABA: gamma-aminobutyric acid

A targeted LC-MS-based metabolomics experiment was performed to determine the impact of blood contamination on the metabolic profile of the samples. The workflow included protein precipitation and extraction of hydrophilic metabolites from ~ 10 mg-aliquots of the homogenates and subsequent parallel HILIC-RP-Orbitrap high-resolution MS. 108 target metabolites were detected in at least 80% of the samples. The samples were grouped into three classes depending on the degree of blood contamination: low (≤ 25 µg/g iron), medium (25–50 µg/g iron), and high (> 50 µg/g iron). A t-test comparing the low- and high-contamination group revealed significantly different signal intensities (mTIC-normalized) for 16 target metabolites with adjusted p-values ≤ 0.05 (Fig. 1). The corresponding scatter plots are depicted in Figure S2. The effect of mTIC normalization is illustrated in Figure S3. 10 of the significantly altered metabolites showed log2 fold-changes ≥ 1. The mean signal intensities were more than doubled for gamma-aminobutyric acid, guanidinoacetic acid and ornithine in the contaminated samples and more than halved for putrescine, pantothenic acid, cytidine, adenine, adenosine, N-acetylputrescine, and fructose-1,6-bisphosphate.

### Unsupervised clustering

**Fig. 2:**
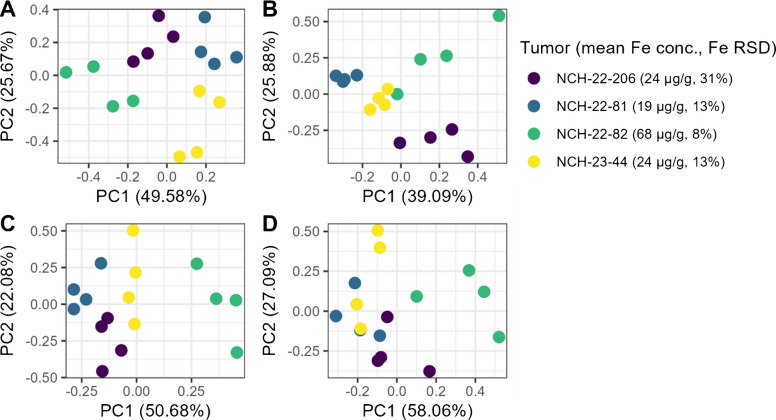
Unsupervised clustering focusing erythrocyte-specific pathways. **A** Anaerobic glycolysis (14 metabolites), **B** purine and nucleoside salvage pathway (18 metabolites), **C** redox metabolism (18 metabolites), **D** pentose phosphate pathway (5 metabolites). 4 tumor samples with 4 replicate samples each

To provide a complete set of data for PCA analysis, missing intensity values were imputed for 2′-deoxycytidine (6 out of 61 samples), 6-phosphogluconic acid (4 samples), flavin adenine dinucleotide (1 sample), fructose-1,6-bisphosphate (1 sample), and bisphosphoglyceric acid (6 samples) and gamma-glutamylcysteine (1 sample). The PCA analysis including all 61 tumor homogenates and a broad panel of target metabolites (mTIC-normalized peak intensities, N = 108) did not result in distinct clustering, and the blood content (approximated by the iron concentration) did not obviously affect the distribution (Figure S4). The clustering of 16 tumor homogenates derived from four patients (N = 4 technical replicates per patient/tumor) including the same broad panel of target metabolites (N = 108) was examined in more detail (Figure S5): The technical replicates from the same patient clustered tightly while the different patients were clearly separated. Technical replicates of tumors with a more homogeneous iron distribution did not cluster more tightly than the technical replicates of a tumor with less homogeneous iron distribution (Figure S5). Notably, the tumors NCH-22–81 and NCH-22–44, which are neighboring in the PCA plot, showed the same distinct tallowy matrix consistency during sample workup. The clustering analysis was repeated with smaller, pathway-specific sets of metabolites focusing on metabolic pathways that are prevalent in erythrocytes as these pathways were considered to be most vulnerable towards blood contamination: pentose phosphate pathway, anaerobic glycolysis, purine and nucleoside salvage pathway, and redox metabolism [27]. The clustering of technical replicates from the same tumor/patient was reduced for the pathways that dominate erythrocyte metabolism (Fig. 2A–D, especially for molecules from the pentose phosphate pathway (5 molecules, Fig. 2D). Focusing on the redox metabolism (18 metabolites), the tumor with the highest iron concentration built a separate cluster, while the tumors with lower iron concentrations clustered together (Fig. 2C). However, there was no distinct association between iron concentration and impact on redox metabolism when all samples were included in the clustering analysis (Figure S5).

### Metabolite quantification

Metabolite concentrations were estimated via external calibration for 75 unique metabolites detected ≥ LLOQ in at least 10 samples via solvent-based external one-point calibration using compound-specific standards. Four pairs of isomers lacking chromatographic separation at baseline were additionally quantified via their summed intensities. Table S4 states the calculated concentrations per sample, Table S5 and Figure S7 give a summary of the measured concentrations. Validation parameters of the quantification based on repeated pooled QC sample injections can be found in Table S6. A list comprising signal intensities before and after mTIC normalization for 108 metabolites is given in Table S7.

## Discussion

In this study, we quantified the extent and distribution of blood contamination in tissue samples obtained during neurosurgery by using iron as a marker for blood. We assessed its potential impact on metabolomics data focused on the hydrophilic central carbon metabolome [11].

Across patients, blood contamination varied markedly, with iron concentrations ranging from 5 to 80 µg/g tissue and few samples reaching ~ 160 µg/g. Given that iron concentrations in whole blood are approximately 500 µg/g, this corresponds to an estimated 1–15% (m/m) blood contamination, with extremes up to ~ 30% (m/m). These values are consistent with the findings of Brem et al., who reported an average blood content of 10± 6% (m/m) in eight meningioma samples [28]. The blood content in the samples was not quantified directly but inferred using iron as a surrogate marker. A broad spectrum of iron concentrations has been described for meningiomas before and was attributed to blood contamination during resection or microbleedings in the meningiomas [29]. However, more recent studies indicate that highly vascularized meningiomas tend to accumulate iron in the cytoplasm of cells neighboring blood vessels [28, 30, 31]. In our study, we reported iron concentrations irrespective of the specific iron species. The reported iron concentrations should therefore be viewed as a rough approximation of the blood and erythrocyte burden in the samples.

During PCA analysis, the metabolic profiles based on the targeted panel of metabolites clearly separated tumors from different patients, while replicate samples from the same tumor clustered tightly. We found no indication that blood contamination was a major factor systematically distorting the overall profile of the targeted metabolite panels. This was true for a metabolite panel consisting of general central carbon metabolism targets as well as for target panels specifically describing the metabolism of erythrocytes. Based on the targeted metabolites, a higher blood content neither masked inter-patient differences, e.g. by dominating the metabolome of more contaminated samples, nor inflated the apparent heterogeneity among samples from the same tumor. Because our analysis was technically limited to hydrophilic metabolites, caution is warranted when generalizing these findings to other metabolite classes—particularly to lipids or redox-active compounds. For instance, Searfoss et al. showed that hemolysis can markedly affect structural lipid concentrations in serum and plasma [1].

An analysis on single metabolite level revealed that elevated iron concentrations were statistically associated with the concentration of 16 metabolites. Blood remains metabolically active ex vivo [26, 32] and the metabolism of tumors with higher vascularization likely suffered more from hemolysis and metabolic adaptation to ischemia. Metabolites that are enriched in tumor tissue like polyamines [33], nucleosides [34, 35], fructose-1,6-bisphosphate (accumulating inside actively glycolyzing cells), creatine [36, 37], serine [38] and threonine [39], are vulnerable to dilution and ex vivo turnover when blood contamination is present, especially in combination with delayed quenching. Nucleosides are actively scavenged by erythrocytes [34, 35], reducing free extracellular adenine and adenosine concentrations. Metabolites enriched in blood cells or generated under hypoxic stress like ornithine (via erythrocyte arginase) [40, 41], gamma-aminobutyric acid and glutamate [42, 43], histidine [44], and guanidinoacetic acid from creatine-pathway imbalance [45] increased. An analysis of the linear correlations of iron concentration and metabolite intensities suggests an overlay of true metabolic alterations between the tumors of different patients and contamination effects. We suggest that future studies targeting these metabolites should critically reflect blood contamination as a biasing factor.

The reported metabolite concentrations should be interpreted as rough estimates, as they do not account for extraction efficiency or matrix effects. Under-quantification is expected for metabolites with low extraction recovery or poor MS ionization efficiency. Furthermore, the time between tumor excision and freezing exceeded one minute and limited warm time during homogenization was unavoidable. Thus, concentrations of degradation-prone metabolites (e.g., inosine, hypoxanthine, xanthine, succinate) are likely overestimated [46]. Nevertheless, we observed high taurine concentrations in agreement with previous reports on meningioma tissue [47]. Despite these limitations, we believe that the presented data will suit as a valuable basis for future metabolite quantification in meningioma [46, 47]. Lastly, this investigation pointed out that meningioma is a complex tissue for metabolomics investigations. Despite careful normalization of the sample amounts based on fresh weight before extraction, we experienced different summed amounts of target metabolites, expressed as the total ion current of the targeted and positively identified metabolites, mTIC. At the same time, the tumor samples appeared not only in different shades of red (indicating different blood/iron content), but also in different textures when they were cut to pieces. The arachnoid layer of healthy meninges mainly consists of fibroblasts and collagen [10]. As metabolically active, living cells, fibroblasts have a rich, dynamic intracellular pool of hydrophilic metabolites. Collagen, on the other hand, is a structural protein produced by fibroblasts. It mainly consists of glycine, proline and hydroxyproline [48] and has low metabolic activity, thus lacking the soluble, low-molecular-weight metabolic intermediates that are analyzed within this metabolomics assay. Additionally, although arising from the middle layer of the meninges (the arachnoid mater), meningiomas are attached to the outer layer of the meninges, the dura mater. The dura mater mainly consists of dense extracellular matrix and harbors blood and lymphatic vessels [49]. Surgical removal of meningiomas often involves resecting part of the dura to prevent recurrence [50]. Some of the analyzed specimens likely contained dura mater tissue.

Against this background, we interpreted our observations, i.e., different textures and differing mTICs, such that the analyzed samples contained different amounts of metabolically active cells despite fresh weight normalization. Normalization to the total ion current of the positively identified metabolites (mTIC) [23, 24] appeared as the most suitable normalization method for this complex situation, accounting for cell number and technical intensity drifts. However, like other normalization methods, mTIC normalization is not free of errors. It assumes that the sum of all detected metabolites is the same in all cells and could otherwise lead to the distortion of metabolic profiles when single metabolites, especially highly abundant ones with major contribution to the mTIC, are metabolically regulated between the samples. We therefore provide raw intensities of our data in Table S7 for complementary data interpretation with more sophisticated statistical tools [51].

## Conclusion

This study systematically evaluated the extent of blood contamination in clinical meningioma tissue and its potential influence on hydrophilic metabolomics readouts. By using iron as a surrogate marker for blood content, we demonstrated that contamination varies substantially between patients but remains relatively consistent within technical replicates of the same tumor. We found that blood contamination was statistically associated with changes in a subset of metabolites but it was not a major factor in unsupervised sample clustering in our specific context. It did not obscure inter-patient metabolic differences or artificially increase intra-tumoral heterogeneity. However, our conclusions regarding the impact of blood contamination on hydrophilic metabolomics are inherently dependent on the analyzed tumor type, the selection of targeted metabolites and the normalization method employed. Nonetheless, the present study creates awareness that blood contamination is an underappreciated factor in tissue metabolomics and that meningioma is a challenging matrix for quantitative metabolomics.

Next to these conclusions, we provide raw intensities and estimated absolute concentrations for a broad panel of metabolites in human meningioma, offering a reference dataset for future quantitative studies. While concentrations should be interpreted with caution due to varying extraction efficiency, matrix effects, normalization effects and potential metabolite degradation during sample handling, our results include relevant real-life metabolite ranges and confirm previously reported high taurine levels in meningioma.

Together, these data establish an empirical basis for handling blood contamination in tissue metabolomics and contribute the first set of reference concentration estimates for the meningioma central-carbon metabolome. The dataset and analytical approach presented here will support improved study design, biomarker discovery, and the development of quantitative metabolomics workflows in clinical neuro-oncology.

## Supplementary Information

Below is the link to the electronic supplementary material.

Supplementary file1 (DOCX 5.29 MB)

Supplementary file2 (XLSX 671 KB)

## Data Availability

The data is made available upon request.

## Declarations

### Source of biological material

*Homo sapiens*. The samples were obtained at the Department of Neurosurgery, Medical University of Vienna.
