# Supplementary material for: Impact of blood contamination on hydrophilic metabolomics in human meningioma tissue
Source: Anal Bioanal Chem. 2026 Jul 1;418(17):5521–30. doi: 10.1007/s00216-026-06623-x (PMC13473324; doi:10.1007/s00216-026-06623-x)
Supplement: Supplementary file 1 [file 216_2026_6623_MOESM1_ESM.docx]

## SUPPLEMENTARY FIGURES

61 tumor samples were obtained from 36 patients (25 were technical replicates (N = 2 or 4) derived from the same patient). Each sample was individually weighed and homogenized and a 10 mg-aliquot was subjected to protein precipitation and metabolite extraction. Four technical replicates (a-d) were obtained for 4 tumors. They were used to assess the intra-tumor heterogeneity of iron concentration and metabolite abundances.

**A**

**B**

Figure S 1 Tumor samples from different patients are contaminated with different amounts of blood. A) Heterogenous coloration of tumor homogenates. Homogenates from three different tumors are depicted for demonstration. B) Iron concentrations measured via ICP-MS. Each circle represents an independently analyzed tumor homogenate.

Figure S 2 Linear relationship of iron concentration and metabolite intensities for metabolite intensities for metabolites that were significantly altered in group comparisons of low (≤ 25 µg/g iron) and high contamination samples (> 50 µg/g iron).

**A**

**B**

Figure S 3 The effect of mTIC normalization on 108 central carbon metabolites. A) Raw intensities, B) intensities after mTIC normalization. The different colors indicate different metabolites.

Figure S 4 Unsupervised clustering based on all 61 tumor homogenates (derived from 36 patients) and 108 central carbon metabolites.

Figure S 5 Unsupervised clustering based on 4 tumors with 4 replicate samples each (108 central carbon metabolites).

Figure S 6 Unsupervised clustering based on all 61 tumor homogenates (derived from 36 patients), focusing on 18 redox metabolies

Figure S 7 Mean and range of central carbon metabolites quantified in meningioma (including technical replicates). A) Amino acid metabolism, B) carbohydrate metabolism, C) cofactors and vitamins, D) energy metabolism, E) nucleotide metabolism, F) lipid and carnitine metabolism, G) polyamine and methylation pathways, H) urea cycle & nitrogen disposal, I) miscellaneous. The dots indicate individual sample concentrations.
